# Comparative evaluation of sequencing technologies and primer sets for mouse gut microbiota profiling

**DOI:** 10.3389/fmicb.2025.1584359

**Published:** 2025-05-20

**Authors:** Aleksandra Strokach, Polina Zoruk, Daria Boldyreva, Maxim Morozov, Evgenii Olekhnovich, Vladimir Veselovsky, Vladislav Babenko, Oksana Selezneva, Natalia Zakharevich, Andrey Larin, Severina Koldman, Vail Koldman, Maya Odorskaya, Roman Yunes, Vladislav Pavlov, Anna Kudryavtseva, Valeriy Danilenko, Ksenia Klimina

**Affiliations:** ^1^Lopukhin Federal Research and Clinical Center of Physical-Chemical Medicine of Federal Medical Biological Agency, Moscow, Russia; ^2^Burnasyan Federal Medical Biophysical Center of Federal Medical Biological Agency, Moscow, Russia; ^3^Vavilov Institute of General Genetics, Russian Academy of Sciences, Moscow, Russia; ^4^Engelhardt Institute of Molecular Biology, Russian Academy of Sciences, Moscow, Russia

**Keywords:** gut microbiota, sequencing technologies, metagenome sequencing, Oxford Nanopore Technologies, Illumina sequencing, microbial diversity, high molecular weight DNA, primer combination

## Abstract

**Background:**

Advancements in sequencing technologies, such as Illumina and Oxford Nanopore Technologies (ONT), have significantly improved microbiome research. However, variations in sequencing platforms, primer selection, and DNA quality may influence microbial diversity assessments, particularly in studies of gut microbiota. This study systematically evaluates these factors in mouse gut microbiota analysis, comparing 16S rRNA gene sequencing and metagenome sequencing (MS) across both platforms.

**Results:**

Our findings highlight the critical influence of primer selection on 16S rRNA sequencing results, with certain primer combinations detecting unique taxa that others miss. Despite these variations in taxonomic resolution, all tested primer sets consistently revealed significant differences between experimental groups, indicating that key microbial shifts induced by bacterial cultures remain detectable regardless of primer choice. A comparative analysis of Illumina and ONT 16S rRNA sequencing revealed notable differences in microbial diversity profiling, with ONT capturing a broader range of taxa. In contrast, MS on both platforms showed a high degree of correlation, indicating that ONT sequencing errors have minimal impact on taxonomic diversity estimations. Furthermore, the type of extracted DNA (high molecular weight vs. standard DNA) had little on microbial diversity outcomes, underscoring the robustness of these sequencing technologies.

**Conclusion:**

These results highlight the advantages and limitations of different sequencing strategies in microbiota research. While 16S rRNA sequencing remains a cost-effective tool for assessing bacterial diversity, MS provides superior taxonomic resolution and more precise species identification. Our study advocates for a hybrid approach that combines multiple sequencing technologies to achieve a more comprehensive and accurate representation of microbial communities.

## Background

In recent decades, advancements in DNA sequencing technologies have transformed microbiome research, providing profound insights into the intricate microbial ecosystems inhabiting various environments. These breakthroughs have significantly contributed to our understanding of microbiota, particularly the gut microbiota (GM), which plays a crucial role in host health and disease.

The human gut microbiome is a diverse and dynamic community of bacteria, viruses, archaea, and microeukaryotes that play a crucial role in maintaining host health. This complex ecosystem influences numerous physiological processes, such as nutrient metabolism and immune system development (Wampach et al., 2017; Hooper and Mac Pherson, 2010; Blaut and Clavel, 2007). Additionally, the GM contributes to colonization resistance, acting as a natural barrier against pathogenic microorganisms by competing for resources and producing antimicrobial compounds (Van Der Waaij et al., 1971; Stecher and Hardt, 2008). Disruptions in the composition of the GM have been increasingly associated with a variety of diseases, including inflammatory bowel diseases, obesity, and cancer (Blaut and Clavel, 2007; Iida et al., 2013). Notably, alterations in the GM can influence cancer progression and response to therapy by modulating the tumor microenvironment, underscoring the essential role of microbiota-host interactions in health and disease (Iida et al., 2013).

Murine models are among the most widely used systems for studying GM due to their physiological and microbial similarities to humans (Lloyd-Price et al., 2016). These models have been instrumental in unraveling the complex interactions between GM and various aspects of health and disease, including metabolic disorders and immune responses, thereby deepening our understanding of the microbiome’s role in these processes and paving the way for the development of targeted therapeutics strategies (Turnbaugh et al., 2007; Round and Mazmanian, 2009).

Traditionally, microbiota composition is analyzed using 16S rRNA gene sequencing, which targets specific variable regions of the gene to assess microbial diversity. This approach has been widely adopted across platforms such as Illumina, which uses short reads, and Oxford Nanopore Technologies (ONT), which enables full-length 16S sequencing through long-read capabilities (Klindworth et al., 2013; Li et al., 2021). While 16S sequencing remains a cornerstone of microbiome research, it provides limited information on functional potential and species-level resolution. MS addresses these limitations by enabling species-and strain-level identification as well as functional annotation. Both 16S and MS approaches can be performed using short read (e.g., Illumina) and long read (e.g., ONT) platforms, each with its own advantages and trade-offs (Callahan et al., 2016b; Jünemann et al., 2017).

Importantly, it is now well established that both DNA extraction protocols and the choice of 16S rRNA gene primers significantly influence MS outcomes. DNA extraction method can bias the representation of certain bacterial taxa, especially those with more resilient cell walls, such as Gram-positive organisms (Galla et al., 2024; Maropola et al., 2015; Sui et al., 2020), while factors like DNA quality, fragmentation, and contaminants can alter the results of metagenomic analysis (Demkina et al., 2023). Likewise, primer selection strongly affects taxonomic resolution and diversity estimates, as some primer sets preferentially amplify specific taxa or exclude others, and certain variable regions (e.g., V3–V4) may not allow for species-level classification (Abellan-Schneyder et al., 2021). Specific primers may also reduce amplification of unwanted host or organellar sequences (Nakano, 2018), or improve archaeal detection (Bahram et al., 2019).

Previous studies have primarily focused on comparing 16S rRNA sequencing results between Illumina and ONT, assessing taxonomic resolution and rare taxa detection while largely overlooking MS data. For example, ONT-based sequencing has demonstrated superior species-level classification and improved detection of rare taxa compared to Illumina in GM studies (Szoboszlay et al., 2023). Similarly, microbial profiling discrepancies have been reported between ONT full-length 16S rRNA sequencing and Illumina V3–V4 region sequencing in head and neck cancer tissues (Yeo et al., 2024). Other studies have examined taxonomic resolution differences between platforms in human nasal microbiota and assessed ONT’s EPI2ME pipeline performance (Heikema et al., 2020). Additionally, some research has investigated the impact of different PCR conditions on sequencing outcomes. Although these studies provide valuable insights, they primarily focus on isolated factors—such as platform performance or PCR parameters—without addressing their combined effects or broader implications for microbiome research (Fujiyoshi et al., 2020).

Our study fills this gap by providing a comprehensive assessment of key methodological variables in MS. We systematically evaluate the impact of primer selection and sequencing platforms on microbial diversity while comparing MS strategies across long-read (ONT) and short-read (Illumina) technologies. Notably, our MS analysis revealed a high degree of correlation between the Illumina and ONT platforms, highlighting their complementarity in microbial composition assessment. Additionally, we examined the influence of different DNA extraction methods on microbial diversity profiling, shedding light on how extraction protocols shape sequencing outcomes. By integrating these factors, our study offers a holistic perspective on microbiome sequencing variability, informing best practices and enhancing the reliability, comparability, and complementarity of different methods. This integrated approach not only evaluates the influence of individual parameters on sequencing outcomes but also reveals their interactions, ultimately informing best practices for microbiome research.

## Methods

### Experimental design

The study was conducted on 27 female C57BL/6 mice obtained from the Stolbovaya Branch of the Federal State Budgetary Institution of Science, “Scientific Center of Biomedical Technologies of the Federal Medical and Biological Agency.” All animals were certified as healthy and possessed veterinary certificates attesting to their health status. Prior to the experiment, a 14-day quarantine period was observed for the animals. Subsequently, they were randomly allocated into three groups: the control group (designated as “Control”), mice administered with lactobacilli (“Lacto” group), and mice administered with bifidobacteria (“Bifido” group). The mice were housed in standard cages (*n* = 9 per cage) maintained at a temperature range of +20–23°C and a humidity level of 60–65%, under natural light conditions with forced ventilation. Sterilized wood shavings served as bedding material. Throughout the duration of the experiment, the mice had ad libitum access to both water and certified briquetted feed.

**Figure 1:**
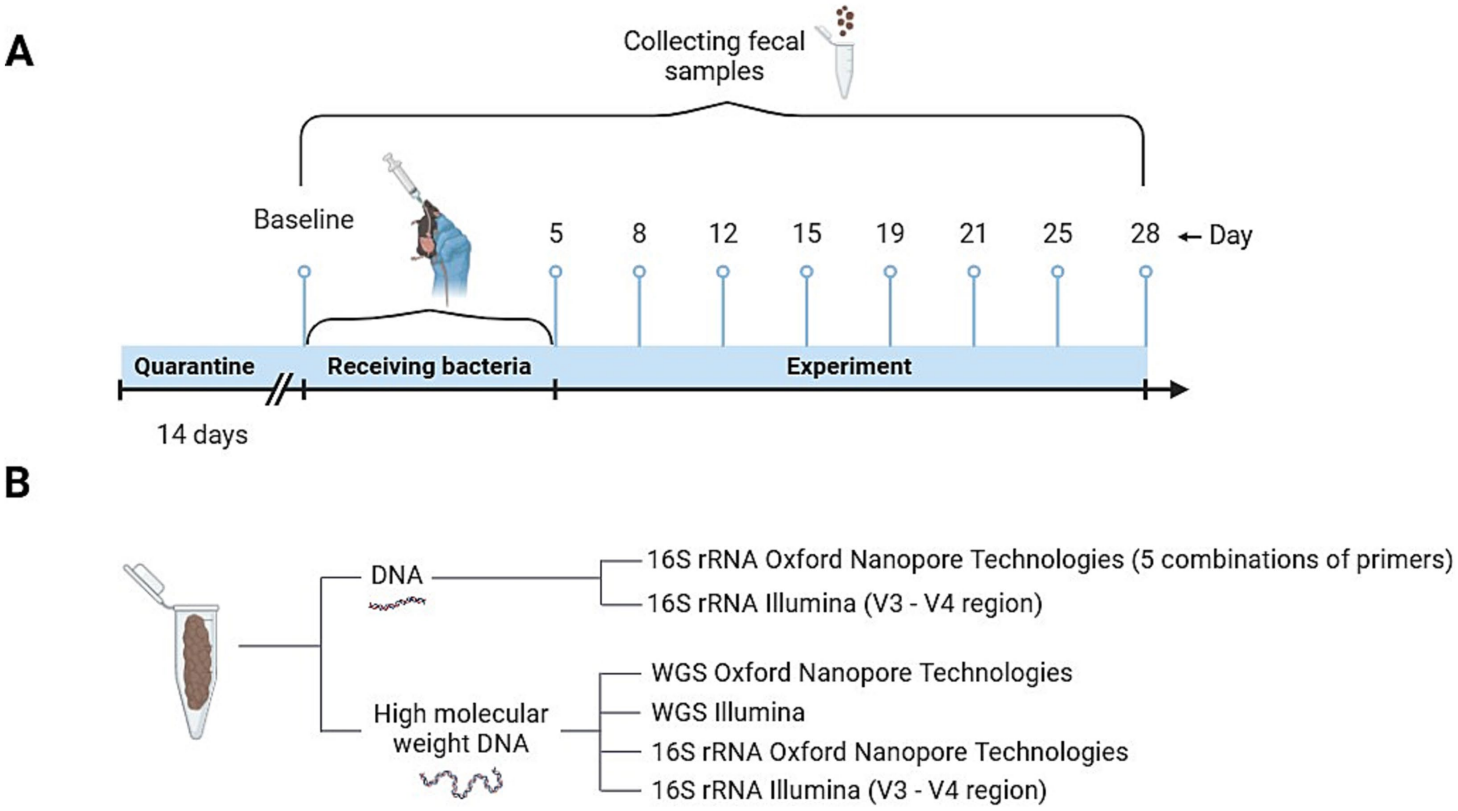
Scheme of experiment. **(A)** Diagram illustrating the design of the experiment on mice treated with bacteria. **(B)** DNA extraction techniques utilized for various sequencing analyses.

During the experiment, each mouse was intragastrically injected with 0.3 mL (10^8 CFUs/ml for *Lacticaseibacillus rhamnosus* K32 and 10^7 CFUs/ml *Bifidobacterium adolescentis* 150) of culture daily for 5 days (Figure 1A). The control group was injected with 0.3 mL of phosphate-buffered saline (PBS). Disposable sterile probes were used for injection.

Fecal samples from mice were collected in 1.5 mL Eppendorf tubes in the morning, following bedding replacement. Feces were taken at days 0, 5, 8, 12, 15, 19, 21, 25, 28 and were stored at −80°C until use. All collected time points were used for the comparison of sequencing platforms and primer combinations (Figure 1B).

### Bacterial strains and growing conditions

The strains *L. rhamnosus* K32 (GenBank accession number JNNV00000000) and *B. adolescentis* 150 (LBHQ00000000) were cultivated for 18 h under anaerobic conditions (10% CO₂ atmosphere, Anaerobic System Mark II, HiMedia, India) at +37°C in Man–Rogosa–Sharpe (MRS) medium (HiMedia, India). For *B. adolescentis* 150, the medium was additionally supplemented with 0.05% cysteine (Dyachkova et al., 2015).

### Lyophilization of cultures

Bacterial cells in the stationary growth phase were harvested and washed with sterile PBS and were then dissolved to a solution composed 1% gelatin and 10% sucrose. The resulting suspension was dispensed into ampicillin vials in 5 mL aliquots. This mixture was incubated for 24 h at −20°C, followed by drying in a 2.5-L Labconco freeze dryer (Labconco, USA) under a pressure of 0.42 mBar and a temperature of −52°C for 48 h. The resulting lyophilized products were stored in vials at +4°C. The lyophilized strains *L. rhamnosus* K32 and *B. adolescentis* 150 were then dissolved in 5 mL PBS before injection. The CFUs/ml of *L. rhamnosus* K32 was 6 × 10^9 before lyophilization and 3.2 × 10^8 after, while for *B. adolescentis* 150, the CFUs/ml counts were 5 × 10^8 before lyophilization and 1.08 × 10^7 after. The difference in CFU concentrations between *L. rhamnosus* K32 and *B. adolescentis* 150 is due to inherent strain-specific growth properties and differences in viability following lyophilization under standardized conditions.

### DNA extraction for 16S rRNA analysis

Nucleic acids were extracted using the PureLink™ Microbiome DNA Purification Kit (Thermo Fisher Scientific, USA). To each of the six stool samples collected from mice, 400 μL of PBS was added. Subsequently, the samples were transferred to bead tubes from the kit for homogenization using MagNA Lyser (Roche, Switzerland). Following homogenization, the samples were centrifuged for 1 min at 7,000 g, after which the supernatant was transferred to a new tube and subjected to extraction using the manufacturer’s protocol. The resulting DNA was then quantified using the Qubit 4 fluorometer with the Quant-iT dsDNA BR Assay Kit (Thermo Fisher Scientific, USA).

### Extraction and purification of high molecular weight DNA

Nucleic acids were extracted using the Wizard Genomic DNA Purification Kit (Promega, USA) with protocol modifications. Six stool samples were transferred to 15 mL tubes, and 2 mL of 50 mM EDTA along with 500 μL of 20 mg/mL Lysozyme (Sigma-Aldrich, USA) were added to each sample. The samples were incubated at a thermal shaker (Allsheng, China) at 37°C 65 g for 1.5 h with intermittent pipetting for homogenization. After incubation, the samples were centrifuged at 3,000 g for 2 min using a Centrifuge 5,804 R (Eppendorf, Germany), and the supernatant was transferred to a new tube. The remaining pellet was resuspended in 1 mL of Nuclei Lysis Buffer, homogenized by pipetting, and incubated in a thermal shaker at 56°C and 300 rpm for 1.5 h. After incubation 400 μL of Protein Precipitation Solution was added and samples were vortexed briefly before being incubated on ice for 10 min. The samples were then centrifuged at 3,700 g for 10 min, and the resulting supernatant was transferred to a new 15 mL tube containing 1.4 mL of isopropanol. The samples were incubated at −20°C for 1 h, followed by centrifugation at 3,700 g for 20 min. The supernatant was discarded, and the DNA pellet was washed twice with 80% ethanol, air-dried for 10–15 min, and resuspended in 200 μL of Low TE buffer.

For additional purification, an equal volume of 2% CTAB solution (100 mM Tris pH 8.0, 20 mM EDTA, 1.4 M NaCl, pre-warmed to 55–65°C) was added to the extracted DNA. The mixture was gently inverted to mix and incubated at 65°C for 10 min. An equal volume of chloroform was then added, vortexed briefly (~30 s), and centrifuged at 3,500 g for 5 min to separate the phases. The upper aqueous layer was transferred to a fresh tube, and 1.1 volumes of 1% CTAB solution (50 mM Tris pH 8.0, 10 mM EDTA, pre-warmed to 55–65°C) was added, mixed thoroughly, and centrifuged at 3,500 g for 5 min, resulting in visible DNA precipitation. The pellet was dissolved in 0.5 mL of 10 mM Tris pH 8.0, 0.1 mM EDTA, and 1 M NaCl by heating at 65°C for 30 min, followed by the addition of 0.6 volumes of isopropanol. The solution was gently inverted multiple times and centrifuged in a microfuge for 8 min. The DNA pellet was washed twice with 0.5 mL of 70% ethanol, air-dried, and resuspended in 100 μL of Low TE buffer, followed by incubation at 65°C for 30 min to ensure complete solubilization. DNA concentration and purity were assessed using a Qubit 4 Fluorometer and a Nanodrop ND-1000 spectrophotometer (Thermo Fisher Scientific, USA).

### 16S rRNA gene sequencing on the MinION platform

The extracted DNA (1–5 ng) was amplified using the forward primers: 27F (AGAGTTTGATYMTGGCTCAG) (Satokari et al., 2002), bif27F (GGGTTCGATTCTGGCTCAG) (Park et al., 2021), and 8F (AGAGTTTGATCCTGGCTCAG) (Turner et al., 1999), in various combinations, with 1492R (GGTTACCTTGTTAYGACTT) (Turner et al., 1999) used as the reverse primer. PCR amplification was performed using the Tersus Plus PCR kit (Eurogen, Russia) in a total reaction volume of 25 μL. Amplification was performed with the following PCR conditions: initial denaturation at 95°C for 2 min, (95°C for 1 min, 60°C for 1 min, and 72°C for 3 min), 27 cycles, followed by a final extension at 72°C for 2 min and 4°C – cooling (Fujiyoshi et al., 2020). The quality of the amplicons was checked by electrophoresis in 1.5% agarose gel. The final amplicons were purified using KAPA HyperPure Beads (Roche, Switzerland) according to the manufacturer’s protocol.

Libraries were prepared according to the manufacturer’s protocol (Ligation sequencing amplicons) with modification. The amplicons were processed with NEBNext® Ultra™ II End Repair/dA-Tailing Module (NEB, USA). Barcodes [Native Barcoding Kit 96 (SQK-NBD109.96) were ligated with Blunt/TA Ligase Master Mix (NEB)]. Barcoded libraries were purified using KAPA Pure Beads (Roche, Switzerland). Library concentrations were measured using the Quant-iT dsDNA Assay Kit, High Sensitivity (Thermo Fisher Scientific, USA) and samples were mixed equimolarity. Final adapter Adapter Mix II Expansion (Oxford Nanopore Technologies, UK) was ligated to the pooled library using the NEBNext Quick Ligation Module (NEB). The prepared DNA library (12 μL) was mixed with 37.5 μL of Sequencing Buffer, 25.5 μL of Loading Beads, loaded onto the R9.4.1 flow cell (FLO-MIN106; Oxford Nanopore Technologies), and sequenced on the MinION Mk1B. MINKNOW software ver. 22.12.7 (Oxford Nanopore Technologies) was used for data acquisition. Reads were basecalled using dorado v.7.6.7 using default parameters [high accuracy (HAC) model, minimum quality value ≥ 7].

### 16S rRNA gene sequencing and analysis on the Illumina platform

For the amplification of extracted DNA (1–5 ng), standard 16S rRNA gene primers targeting the V3-V4 region and incorporating 5’-Illumina adapter sequences (16S Amplicon PCR Forward Primer: 5’ TCGTCGGCAGCGTCAGATGTGTATAAGAGACAG CCTACGGGNGGCWGCAG; 16S Amplicon PCR Reverse Primer: 5’ GTCTCGTGGGCTCGGAGATGTGTATAAGAGACAGGAC TACHVGGGTATCTAATCC) were used. These primers were obtained from Evrogen (Russia). PCR amplification was performed with the Tersus Plus PCR kit (Evrogen, Russia) in a total volume of 25 μL. Library preparation and sequencing on the Illumina platform were performed as described in our previous work (Larin et al., 2024). DNA libraries were sequenced on a MiSeq instrument (Illumina, USA) using the 500-cycle MiSeq reagent kit v2 (Illumina, USA). The results of 16S rRNA gene sequencing were processed and analyzed using a standardized workflow. Fastp v0.23.4 was used for trimming low-quality and filtering of technical sequences (Chen et al., 2018). Processed sequences were analyzed using the DADA2 pipeline v1.26.0 (Callahan et al., 2016a) with taxonomic classification based on the SILVA database v138 (Quast et al., 2013). Differences between sequencing technologies were assessed using LefSe (Segata et al., 2011) from microbiomeMarker package (v1.10.0).^1^

### Bioinformatics analysis of 16S rRNA sequencing data from Oxford Nanopore

Raw sequencing reads were processed using Porechop v 0.2.4^2^ to remove adapters, with default parameter settings. Quality filtering was performed using Chopper v 0.6.0 (De Coster and Rademakers, 2023), applying a Phred score threshold of 10 and selecting sequences within a length range of 100 to 1,800 bp. Taxonomic classification was conducted using the Emu pipeline v 3.4.5 (Curry et al., 2022), while NanoStat v 1.6.0 (De Coster and Rademakers, 2023) was employed to generate read quality statistics. The processed data were imported into RStudio (version 2023.12.0 + 369, R 4.3.2) for further analysis using the MicrobiotaProcess package v 1.16.1 (Xu et al., 2023). Alpha diversity was assessed using Shannon indices, with statistical comparisons performed via the Wilcoxon rank-sum test. Statistical significance between experimental groups was determined using distance-based permutational multivariate analysis of variance (mp_adonis function), with a threshold of *p* < 0.05 after 10,000 permutations. Differential abundance analysis was conducted using the mp_diff_analysis function, with visualizations generated via mp_plot_diff_res, and mp_plot_diff_cladogram (Segata et al., 2011). The ComplexUpset package v1.3.3 (Lex et al., 2014) was used to generate an Upset plot, while additional visualizations were created using ggplot2 v3.5.1 and gplots v3.2.0 (Wickham, 2016). The raw data have been deposited in the NCBI GenBank database: BioProject accession PRJNA1069621 (the 16S rRNA sequences of the mice fecal microbiota).

### Comparative analysis of 16S rRNA gene sequencing using Illumina and ONT

Illumina V3-V4 16S rRNA sequencing data were processed using the DADA2 pipeline (v1.26.0) (Callahan et al., 2016a), with taxonomic classification performed against the SILVA database 138 (Quast et al., 2013). For ONT full-length 16S rRNA sequencing data, the Emu pipeline v3.4.5 was used with the same reference database to ensure consistency in taxonomic assignment (Curry et al., 2022).

### Library preparation and sequencing for Illumina

100 ng of the extracted DNA was used for library preparation using the KAPA HyperPlus Kit (Roche, Switzerland) according to the manufacturer’s protocol. The library underwent a final cleanup using the KAPA HyperPure Beads (Roche, Switzerland) after which the library size distribution and quality were assessed using a high sensitivity DNA chip (Agilent Technologies). Libraries were subsequently quantified by Quant-iT DNA Assay Kit, High Sensitivity (Thermo Fisher Scientific). The DNA libraries underwent sequencing using the HiSeq 2,500 platform (Illumina, USA), in accordance with the manufacturer’s recommendations. For this purpose, we employed the following reagent kits: HiSeq Rapid PE Cluster Kit v2, HiSeq Rapid SBS Kit v2 (200 cycles), and HiSeq Rapid PE FlowCell v2. Additionally, a 2% PhiX spike-in control was included in the process. The raw data have been deposited in the NCBI GenBank database: BioProject accession PRJNA1070000 (metagenomic sequences of the fecal microbiota of mice).

### Metagenomic sequencing on PromethION platform

DNA (1 μg) was used to prepare libraries for ONT according to the manufacturer’s protocol. The long reads were generated with PromethION sequencing (Oxford Nanopore Technologies, UK). The sequencing libraries were prepared using the ligation sequencing kit SQK-LSK109, native barcoding expansion kit EXP-NBD196 and run in a R9.4.1 (FLO-PRO002) flow cell. Reads were basecalled using Guppy v6.5.7 using default parameters (high accuracy (HAC) model, minimum quality value > 7).

### Bioinformatics analysis of Illumina and ONT metagenomic sequencing data

Oxford Nanopore reads were pre-processed in a similar way as 16S data. Illumina metagenomic sequencing data was processed as follows. Quality control of Illumina reads was performed using FastQC.^3^ Fastp v0.23.4 tool was used for removing adapters and low-quality sequences from raw data (Chen et al., 2018). Removing host reads from trimmed data was performed using HiSAT2 v2.2.1 aligner (Kim et al., 2019) and mice genome version GRCm39.^4^ For comparison of different sequencing technologies, Kraken2 v2.1.3 tool with default bacterial database (assembled in November 2022) was used for taxonomic annotation of 16S and MS Illumina and Oxford Nanopore pre-filtered reads (Lu et al., 2022). The resulting taxonomic tables were filtered by summary relative abundance > 0.01%.

## Results

### Assessment of microbial consistency in mouse gut microbiota using diverse 16S rRNA primer pairs

**Figure 2:**
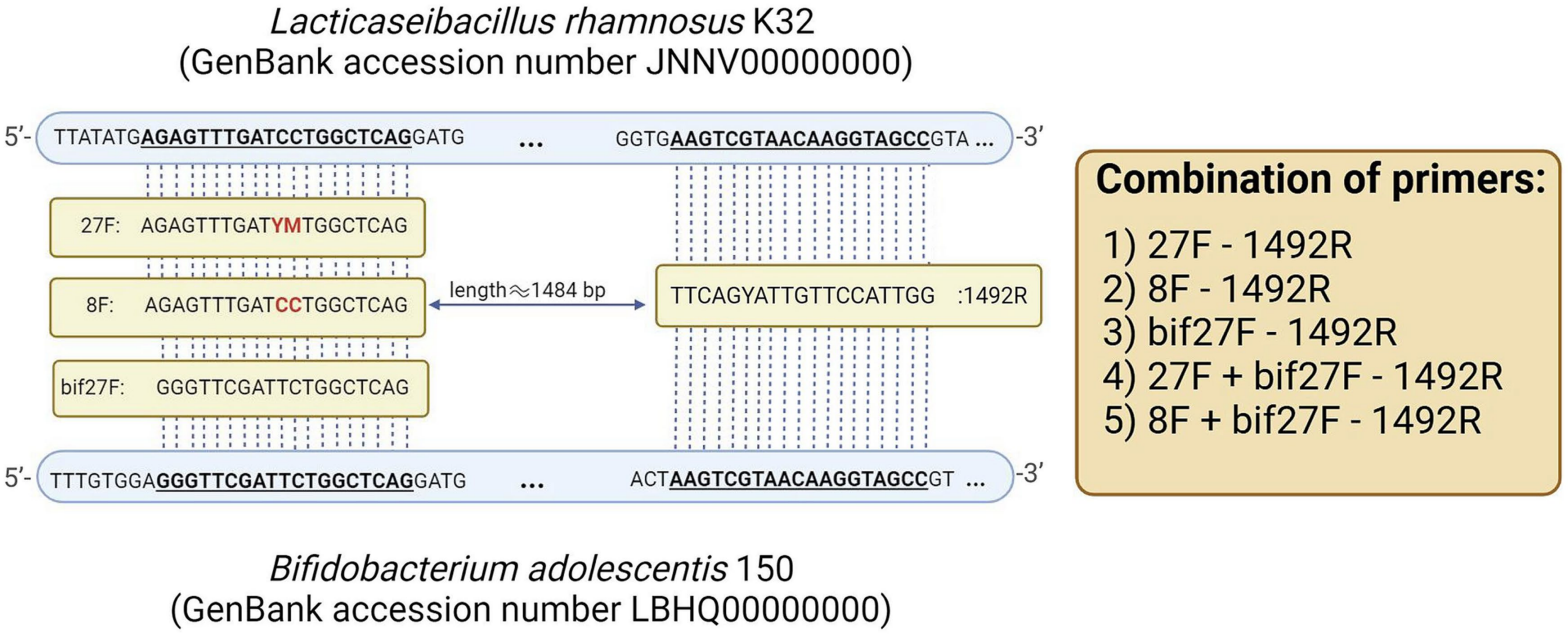
16S rRNA gene with primer binding sites used in this study.

To assess the impact of primer selection on metagenomic analysis, we tested five distinct 16S rRNA primer sets: (1) 27F - 1492R, for broad bacterial profiling; (2) 8F - 1492R, targeting bacterial 16S rRNA genes without degenerate positions; (3) bif27F - 1492R, designed to amplify *Bifidobacterium* species; (4) 27F + bif27F - 1492R and (5) 8F + bif27F - 1492R incorporating a *Bifidobacterium*-specific primer to enhance detection of this genus alongside general bacterial populations. Detection of *Bifidobacterium* species in metagenomic samples can be challenging due to mismatches between universal primers and the 16S rRNA gene sequences of this genus. For instance, the commonly used 27F primer has a known mismatch in the binding region—specifically, a G-to-A substitution frequently found in *Bifidobacterium* (Figure 2).

**Figure 3:**
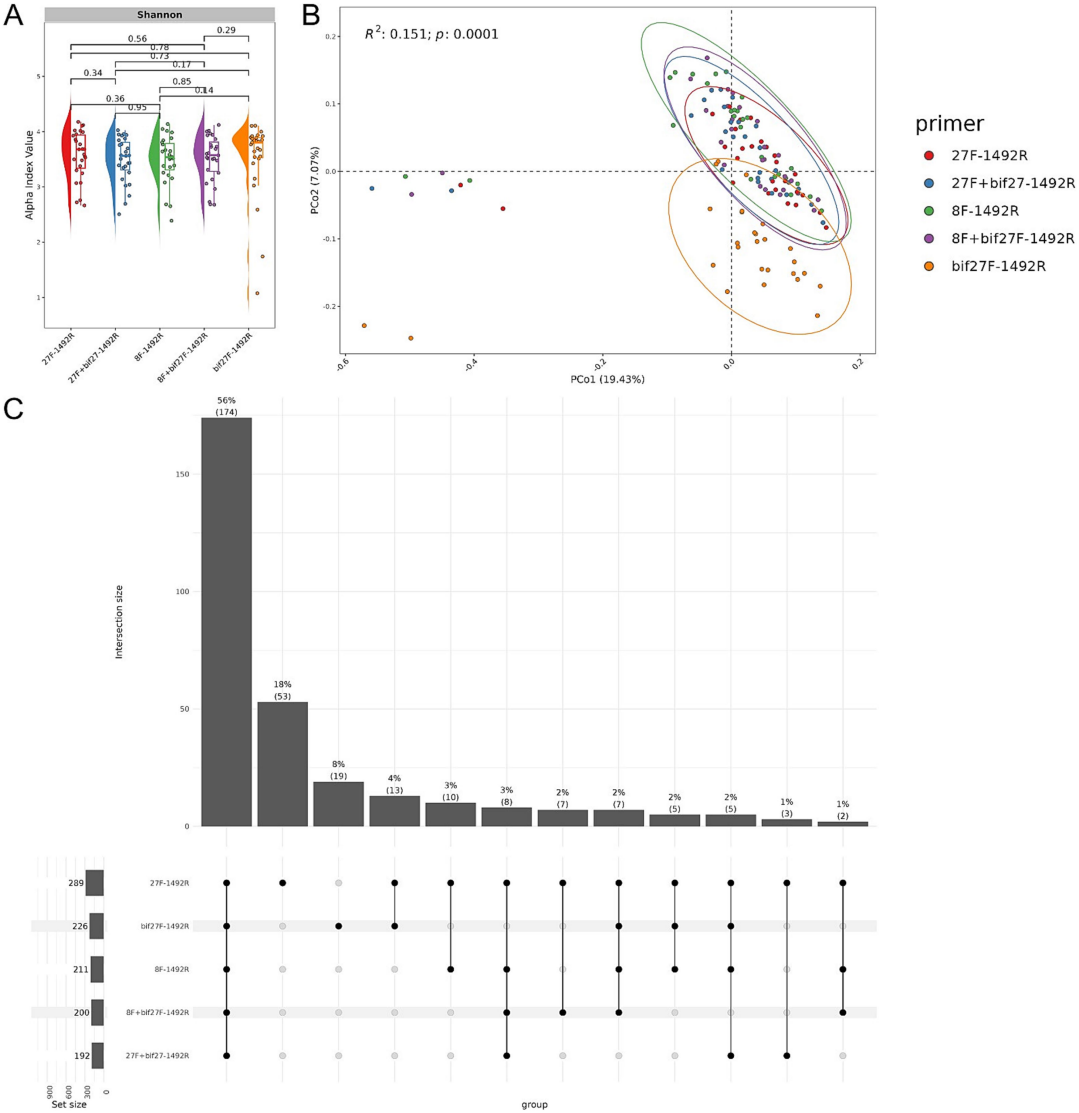
Microbial community analysis in experimental mice via full-length 16S rRNA gene sequencing. **(A)** Raincloud plot of the alpha diversity index. Primer pairs are displayed along the x-axis, with the Shannon index represented on the y-axis, color-coded by primer. **(B)** PCoA plot illustrating microbial community composition. Each point represents a sample, plotted using Bray-Curtis distances, with colors indicating group classifications. The distinct cluster on the left corresponds to baseline microbiota profiles collected before bacterial culture administration. **(C)** UpSet plot showing unique and shared bacterial species across primer sets. Bars indicate the number of species detected by each primer combination.

DNA extracted from the fecal samples of 27 mice underwent full-length 16S rRNA sequencing using the ONT platform, resulting in a total of 2,136,829 reads. After filtering, 1,944,663 high-quality reads were retained, with sequencing distribution details provided in Supplementary Figure S1 and Supplementary Table S1. Despite variations in primer pairs, statistical analysis of alpha diversity confirmed consistent microbial composition across groups (Figure 3A), suggesting minimal impact of primer choice on overall GM diversity in mice. However, beta diversity analysis via PCoA revealed distinct grouping patterns depending on the primer combination. The ‘27F - 1492R’, ‘27F + bif27-1492R’, ‘8F - 1492R’, and ‘8F + bif27F - 1492R’ sets cluster closely together, indicating similar microbial detection profiles (Figure 3B). In contrast, the ‘bif27F - 1492R’ combination formed a distinct cluster, likely dues to its preferential amplification of specific taxa that are less efficiently targeted by universal primers (Supplementary Figure S2). While this primer set did not detect the highest number of unique species, its clustering pattern suggests it may complement general primers by capturing specific groups within the GM.

An UpSet plot analysis illustrated the distribution of unique and shared bacterial species across different primer combinations (Figure 3C). Combining all five primers detected 174 common species, accounting for 56% of all identified species, indicating that the multi-primer approach provides broad coverage. Examining individual primer sets, ‘27F - 1492R’ and ‘bif27F - 1492R’ detect 53 (18%) and 19 (8%) species, respectively. However, when the ‘bif27F - 1492R’ primer is removed from the analysis, the intersection drops to only eight species (3%). This reduction in the common intersection suggests that the other primers detect largely distinct subsets of the bacterial community with relatively little overlap among themselves. In other words, its removal exposes the limited shared species among the remaining primer sets, emphasizing that each primer tends to capture a different facet of the bacterial diversity. Due to the distinct sequence profiles generated by ‘bif27F - 1492R’ primers and its divergence from other primer sets, it was excluded from further analysis to maintain dataset consistency.

**Figure 4:**
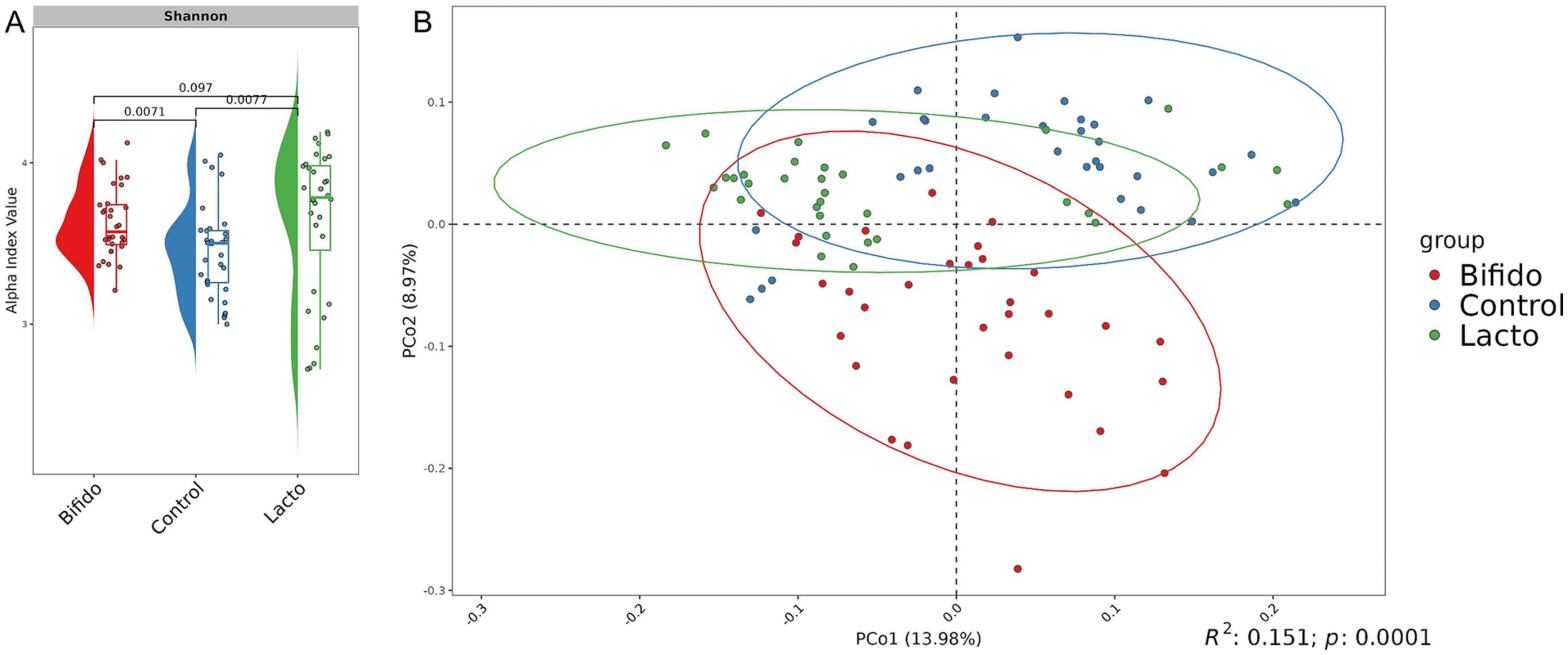
Microbial community analysis of experimental groups. **(A)** Raincloud plot of the alpha diversity index. The x-axis represents the experimental groups (Control, Bifido, and Lacto), while the y-axis represents the alpha diversity index (Shannon). Colors indicate the different groups. **(B)** Bray-Curtis distance-based PCoA plot for each group. Each point represents a single sample, with colors corresponding to group names. The first and second components are shown.

Since our study included three distinct experimental groups (see Materials and Methods), the use of four selected primer combinations allowed us to observe clear group-specific differences in microbial composition. Statistical analysis using the Shannon index revealed significant differences between the control group and the groups receiving lyophilized bacterial cultures, with an increase in alpha diversity observed in the experimental groups. This suggests a potential beneficial effect on the intestinal microbiota (Figure 4A). Beta diversity analysis further demonstrated that the microbiota structure significantly differed between experimental groups (PERMANOVA adj.*p* < 0.0001, 10,000 permutations, Bray–Curtis dissimilarity metric) (Figure 4B).

### Comparative evaluation of 16S rRNA gene sequencing across Illumina and ONT

**Figure 5:**
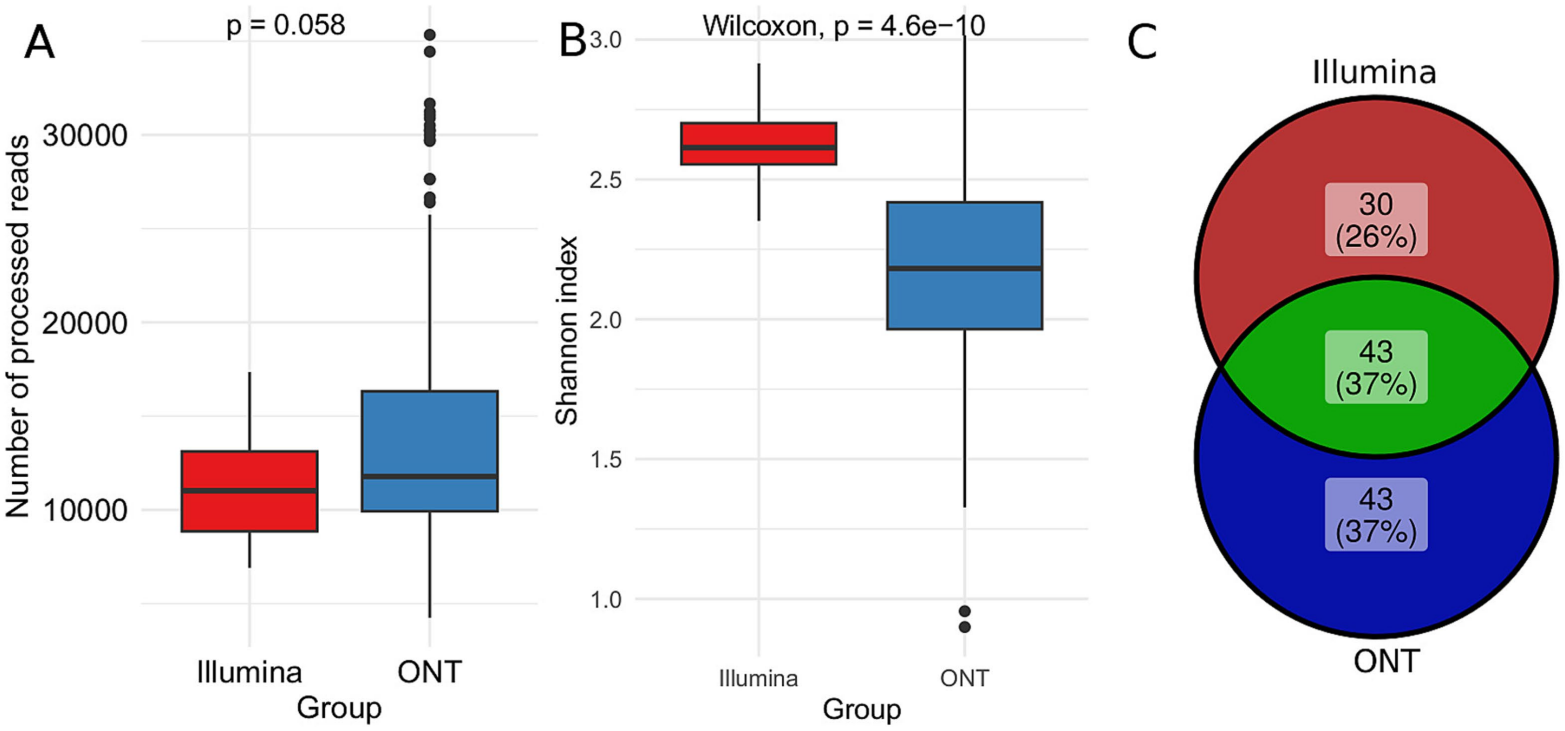
Comparative taxonomic analysis using Illumina and ONT sequencing. The DADA2 pipeline for Illumina data Emu pipeline for ONT data and SILVA database were used for analysis. **(A)** Boxplots illustrating the distribution of read counts accross different sequencing technologies **(B)** Boxplot of the Shannon diversity index, comparing microbial diversity captured by each platform. The Wilcoxon rank-sum test was applied to assess statistical differences **(C)** Venn diagram depicting the overlap of genera identificed by Illumina and ONT sequencing.

To assess differences in microbial community profiling between Illumina and ONT sequencing platforms, we compared their taxonomic diversity outputs. Despite a similar read count (Figure 5A), we observed considerable variation in the Shannon index, which measures genus diversity within the community (Figure 5B). Illumina sequencing identified 73 genera, whereas ONT sequencing identified 86. The Venn diagram (Figure 5C) shows that 43 genera (37%) were detected by both platforms. Additionally, our findings show that Illumina sequencing predominantly identifies *Bacteroides* and related bacteria, whereas ONT sequencing captures a broader range of *Firmicutes* (Supplementary Figure S3).

### Comparison of sequencing technologies for mouse microbiota analysis

**Figure 6:**
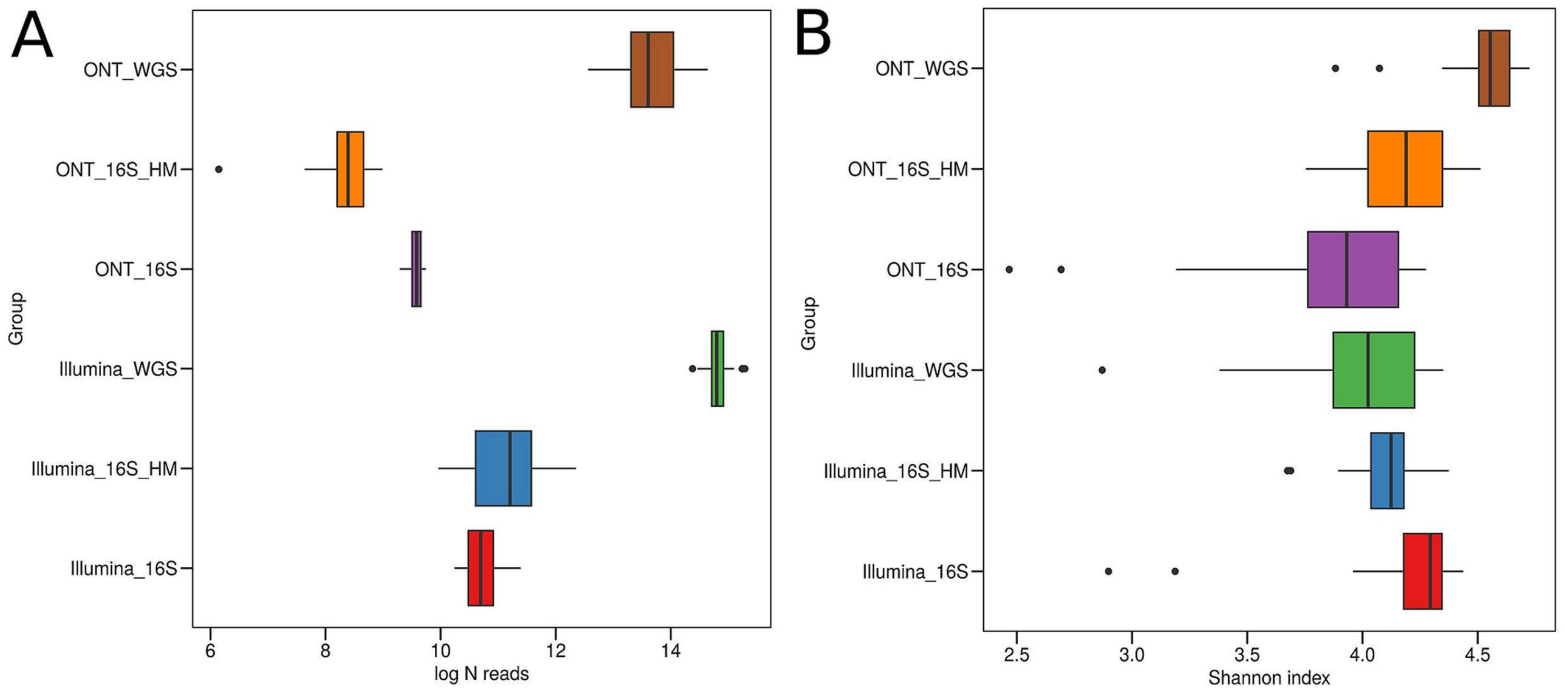
Read count and microbial diversity assessment for different sequencing methods. **(A)** Number of reads obtained from each sequencing approach. **(B)** Shannon index comparing microbial diversity across different sequencing methods.

To compare different sequencing approaches for mouse microbiota analysis, we utilized both high-molecular-weight (HM) DNA and DNA extracted using standard methods (Figure 1B). MS was performed on both Illumina and ONT platforms, with results labeled as Illumina_WGS and ONT_WGS, respectively. Additionally, the V3-V4 region of the 16S rRNA gene was sequenced using the Illumina MiSeq platform for both standard and HM DNA (Illumina_16S and Illumina_16S_HM, respectively). Full-length 16S rRNA gene sequencing was conducted on the ONT platform for both DNA types (ONT_16S and ONT_16S_HM, respectively). To ensure consistency, bacterial diversity assessments were performed using libraries prepared from the same extracted DNA across all sequencing methods. The number of reads obtained from each sequencing approach is summarized in Figure 6A. The average read lengths observed for each sequencing approach were as follows: Illumina_WGS – 197.3 bp, ONT_WGS – 5332.8 bp, Illumina_16S – 501.5 bp, Illumina_16S_HM – 501.6 bp, ONT_16S – 1445.5 bp, and ONT_16S_HM – 1470.7 bp.

MS using both Illumina and ONT platforms (ONT_WGS, Illumina_WGS) yielded the highest read counts. Conversely, full-length 16S rRNA gene libraries derived from HM DNA sequenced on the ONT platform (ONT_16S_HM) had the lowest read counts. Despite this, bacterial diversity remained relatively high, suggesting that ONT sequencing effectively captures bacterial diversity even with fewer reads (Figure 6B). However, when sequencing the 16S rRNA gene from more fragmented DNA (ONT_16S), the results obtained with ONT tend to be less comprehensive compared to those achieved with Illumina.

**Figure 7:**
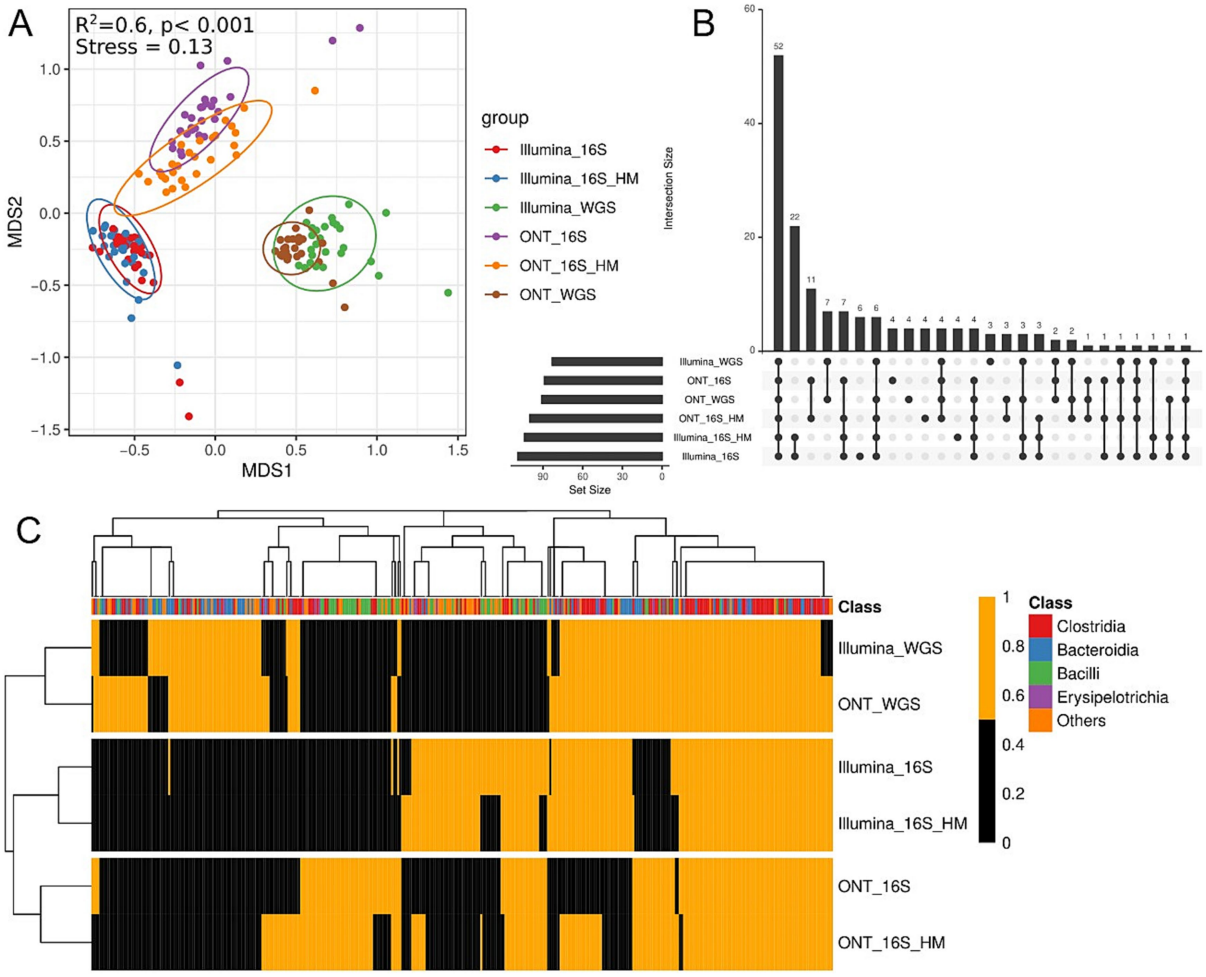
Taxonomic annotation comparisons across sequencing methods. **(A)** Non-metric multidimensional scaling (NMDS) biplot displaying taxonomic profiles across sequencing methods. Different colors indicate different sequencing technologies. The top left graph presents the PERMANOVA test results. **(B)** UpSet showing unique and shared bacterial genera across sequencing methods. Bars represent the number of species detected for each sequencing method. **(C)** Heatmap displaying microbial classes presence/absence based on Kraken2 annotation. Orange indicates presence, black indicates absence. The x-axis represents microbial classes, while the y-axis denotes different sequencing technologies. The top color bar denotes taxonomic classification at the class level. Clustering was performed using Euclidean distance and complete linkage.

Next, we analyzed the similarity in metagenomic composition among samples sequenced using different technologies (Figure 7). Clustering analysis based on sequencing platform revealed a statistically significant correlation between taxonomic class detection and sequencing technology (Figure 7A). Across all platforms, 68 species were identified, but some were unique to specific sequencing methods (Figure 7B). For example, bacteria of the *Bacilli* class are more effectively identified using 16S rRNA methods, whereas certain *Bacteroidetes* were more effectively identified via MS (Figure 7C). A higher number of *Bacilli* genera were observed by 16S rRNA gene analysis (full-length and V3–V4) compared to MS. Furthermore, results obtained using the same sequencing methods for both HM and standard DNA exhibited strong similarity.

**Figure 8:**
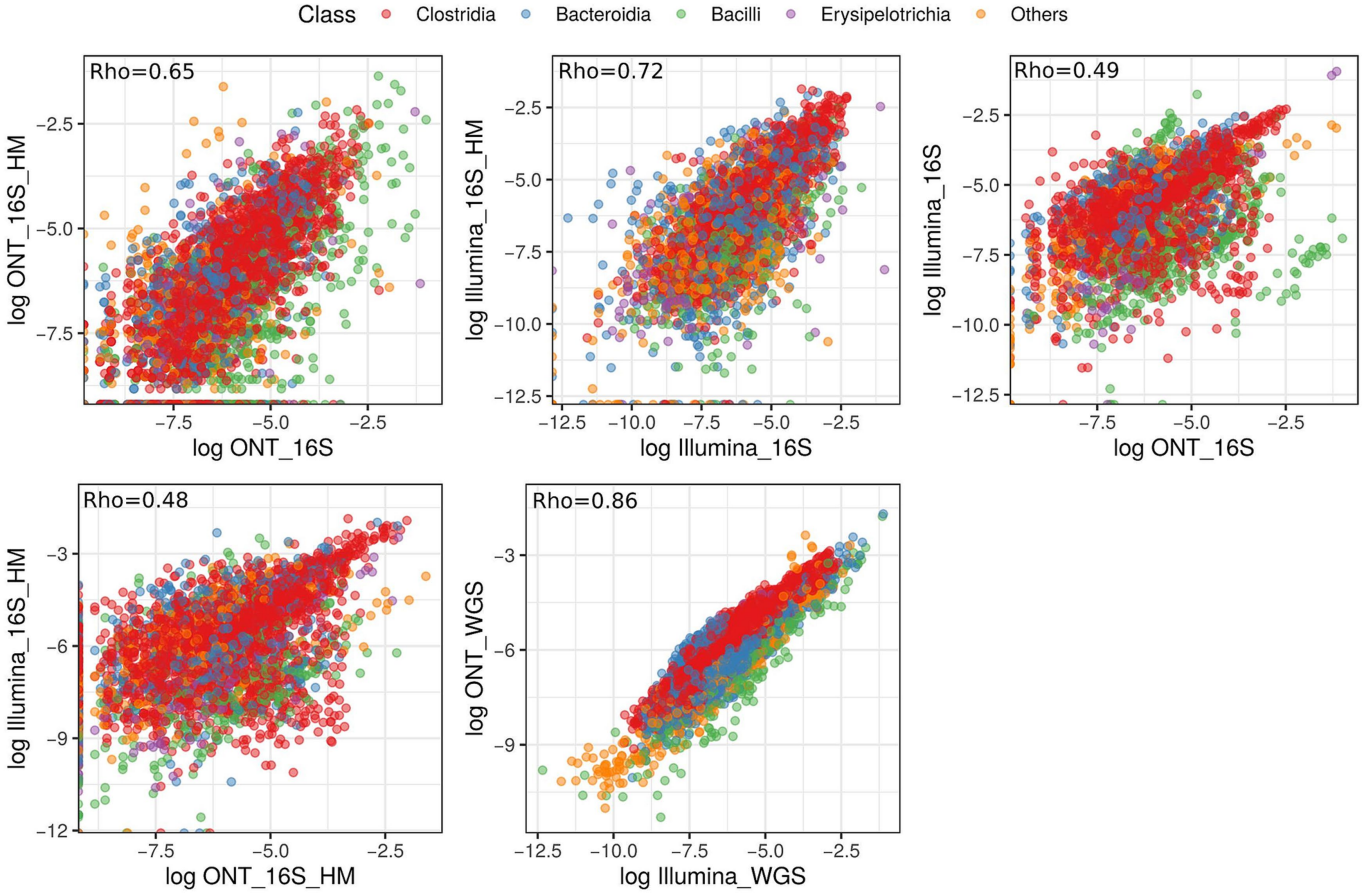
Scatterplot illustrating the correlation in relative abundance values of annotated microbial classes across different sequencing types. Each color represents a distinct taxonomic annotation at the class level. The correlation coefficient is displayed in the upper left corner of each graph.

In our study, we performed pairwise comparisons of microbial composition derived from different sequencing technologies, considering the quality of extracted DNA (Figure 8). Our results showed a strong correlation within each sequencing technology, though variability was influenced by the initial DNA quality. A detailed comparison of 16S rRNA gene sequencing data from illumina and ONT platforms revealed only a moderate correlation between the two (Rho = 0.49 and Rho = 0.48, respectively). In contrast, MS results from Illumina and ONT exhibited a high degree of similarity (Rho = 0.86), suggesting that combining different sequencing approaches may enhance the detection and characterization of bacterial diversity in biological samples.

## Discussion

The comparative analysis of five primer combinations revealed a significant impact of primer choice on the 16S rRNA profiling of mouse GM using ONT sequencing. The PCR conditions used in this study were based on a previously optimized protocol (Fujiyoshi et al., 2020). While all primer combinations exhibited similar alpha diversity, the ‘bif27F - 1492R’ combination notably identified a greater number of unique species. This variability may stem from variations in the GC content of target species, potentially introducing biases in sequencing outcomes (Park et al., 2021). The distinct results obtained with the ‘bif27F - 1492R’ primers, comnpared to others, suggest a limitation in fully capturing bacterial diversity within the samples. Nonetheless, these primers are particularly effective at detecting specific bacteria of interest, such as *Catabacter hongkongensis* — a Gram-positive anaerobic coccobacillus associated with gastrointestinal diseases and potential acute intestinal conditions (Lau et al., 2012; Lau et al., 2007; Valentin et al., 2022). Furthermore, the identification of *Acetatifactor muris* with these primers underscores its potential role in affecting obesity or inflammation through changes in microbiota composition, highlighting the clinical significance of precise microbial profiling in therapeutic settings (Lee et al., 2019). However, while primer selection influences the detection of specific taxa, our results indicate that any chosen primer pair still provides a representative view of the experimental groups. Both alpha and beta diversity analyses revealed significant differences in microbiota composition between groups, suggesting that the key microbial shifts induced by bacterial cultures remain detectable regardless of the primer set used.

Further analysis of 16S rRNA gene sequencing data from both Illumina and ONT platforms revealed significant differences in sensitivity and specificity, particularly in microbiota composition analysis. These differences predominantly stem from the distinct read lengths utilized by each platform. Illumina sequencing targets the V3-V4 (~460 bp) region of the 16S rRNA gene, constrained by its read length limitations, whereas ONT has the capability to sequence the full-length gene (~1,480 bp). This capacity to capture full-length 16S rRNA genes endows ONT with the potential to furnish a more nuanced and comprehensive bacterial profile through sequence alignment with databases, thereby offering a broader and potentially more precise portrayal of bacterial communities within the same sample. Although ONT has a higher per-read error rate, this has limited impact on diversity estimates, as relative abundance patterns — rather than single-read accuracy — play a more critical role in alpha and beta diversity analyses. Moreover, recent improvements in ONT chemistry (e.g., Q20 + with R10.4.1 flow cells) have significantly enhanced sequencing accuracy (Yoon et al., 2017), making ONT increasingly suitable for species-level classification, detection of rare taxa, and richness estimation (Szoboszlay et al., 2023).

A comparative study investigating 16S rRNA gene sequencing of human nasal microbiota across ONT and Illumina platforms found that diversity profiles at the genus level exhibited strong similarities between both platforms. However, ONT yielded significantly fewer *Corynebacterium* compared to Illumina, potentially due to primer mismatches during nanopore sequencing (Heikema et al., 2020). Similarily, Teahyen Cha et al. (2023) examined infant GM that taxonomic profiles identified by ONT closely paralleled those detected by Illumina at the genus level, further supporting ONT’s reliability in identifying bacterial genus identification.

Analyzing sequencing data from 16S rRNA and MS across both Illumina and ONT platforms underscores the value of integrating diverse sequencing methodologies to enhance the resolution of intestinal microbiota composition. In metagenomic analysis, the generation of longer contigs plays a crucial role in achieving high-quality results, which are foundational for various downstream applications. These applications include but are not limited to taxonomic assignments (Patil et al., 2011; Ciuffreda et al., 2021), gene annotation, operon identification (frequently surpassing 10 kb in length), and detecting structural variations (Ye et al., 2022). Notably, initial DNA quality appears to have minimal impact on sequencing outcomes. While MS provided a more detailed view of microbial diversity than 16S rRNA gene sequencing (Brumfield et al., 2020; Lewis et al., 2021), its effectiveness in characterizing low-abundance microbial populations is limited. MS often prioritizes highly represented species, potentially overlooking less prevalent taxa. Although increasing metagenomic read coverage could mitigate this issue, it remains economically impractical. Conversely, 16S amplicon sequencing is more resource-efficient, requiring fewer reads while capturing the “long tail” of relative abundance distributions. However, despite its advantages, 16S rRNA gene sequencing may suffer from reduced taxonomic resolution and primer-induced biases. Our study advocates for a hybrid approach, combining multiple sequencing techniques to achieve a more comprehensive understanding of microbial community diversity, acknowledging that taxonomic identifications can vary depending on sequencing technology. While recognizing potential limitations, such as misclassification with tools like Kraken2 (Lu et al., 2022), existing literature supports the utility of this method for taxonomic annotation across Illumina and ONT platforms using 16S rRNA gene sequencing and MS data (Curry et al., 2022; Lu and Salzberg, 2020; Matsuo et al., 2021).

## Conclusion

In this study, we conducted a comprehensive comparison of Illumina and ONT sequencing platforms to evaluate their performance in profiling mouse GM using 16S rRNA gene sequencing and metagenomic approaches. Our results demonstrate that the choice of primer pairs for 16S rRNA gene sequencing on the ONT platform does not significantly affect microbial diversity, though specific primers can identify unique taxa. Comparative analysis between ONT and Illumina platforms revealed differences in microbial diversity profiling, which are largely attributed to the difference in amplicon length rather than the sequencing platform itself, as ONT’s ability to sequence the full-length 16S rRNA gene provides higher taxonomic resolution compared to the partial V3–V4 region targeted by Illumina. Additionally, MS showed a high degree of correlation between the two platforms, emphasizing their complementarity in assessing microbial composition. Importantly, the quality of DNA (high molecular weight versus standard DNA) did not substantially impact microbial diversity outcomes, highlighting the robustness of these sequencing technologies.

## Data Availability

The datasets presented in this study can be found in online repositories. The names of the repository/repositories and accession number(s) can be found in the article/Supplementary material.

## Footnotes

^1^ https://github.com/yiluheihei/microbiomeMarker

^2^ https://github.com/rrwick/Porechop

^3^ https://www.bioinformatics.babraham.ac.uk/projects/fastqc/

^4^ https://www.ncbi.nlm.nih.gov/datasets/genome/GCF_000001635.27

## Supplementary material

SUPPLEMENTARY FIGURE S1 Number of reads for different primer pairs.

SUPPLEMENTARY FIGURE S2 Bacterial species uniquely identified by the primer combination “bif27F - 1492R” compared to other primers using LefSe.

SUPPLEMENTARY FIGURE S3 Differential taxonomic abundance analysis between 16S Illumina and ONT sequencing using LefSe. Red colors represent taxa identified by Illumina 16S rRNA gene sequencing, while blue colors denote taxa identified by ONT sequencing.

SUPPLEMENTARY TABLE S1 Reads information.
