# Supplementary figures and images for: Comparative evaluation of sequencing technologies and primer sets for mouse gut microbiota profiling
Source: Front Microbiol. 2025 May 20;16:1584359. doi: 10.3389/fmicb.2025.1584359 (PMC12130029; doi:10.3389/fmicb.2025.1584359)

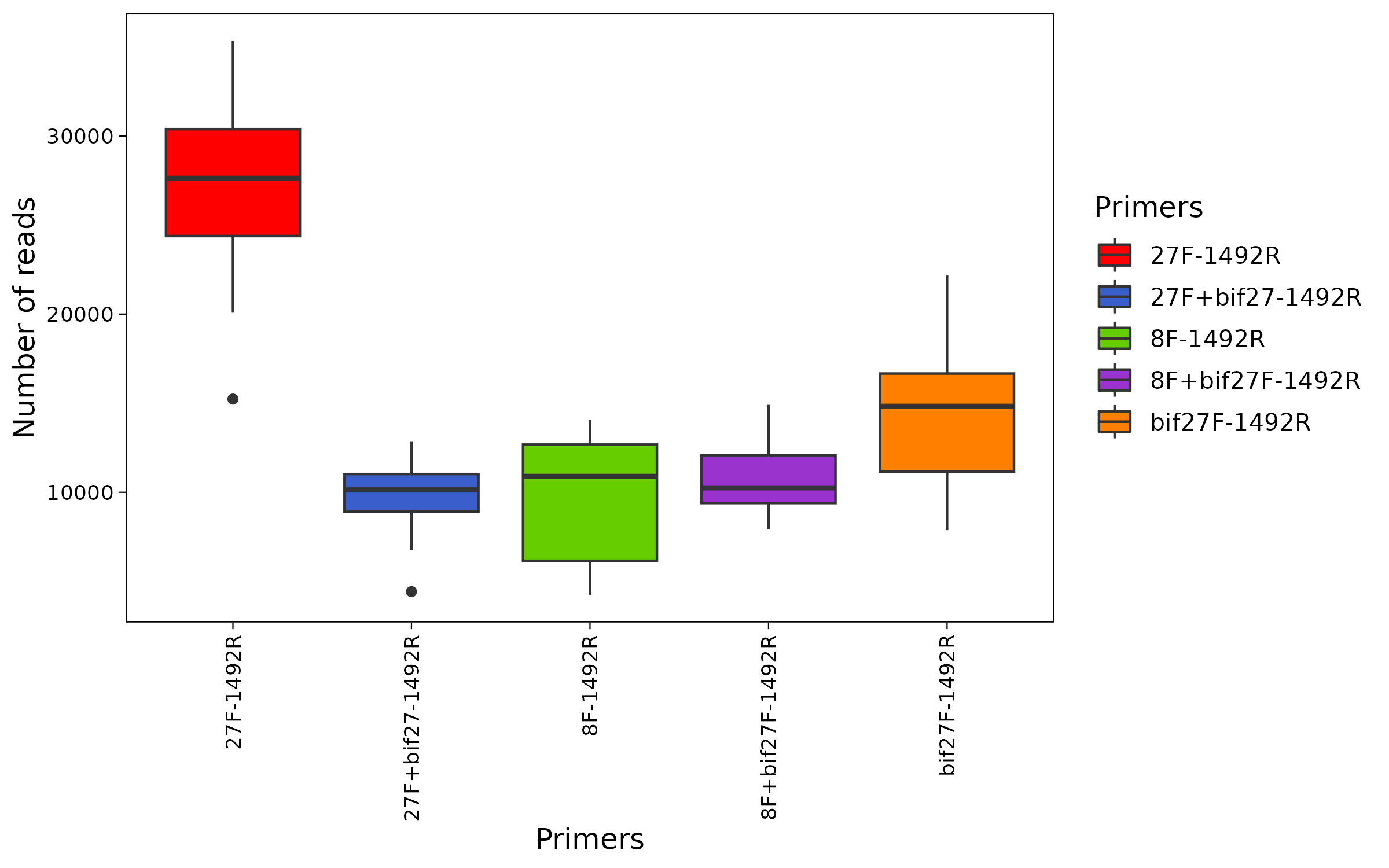

Supplement: SUPPLEMENTARY FIGURE S1 — Number of reads for different primer pairs. [file Image_1.png]

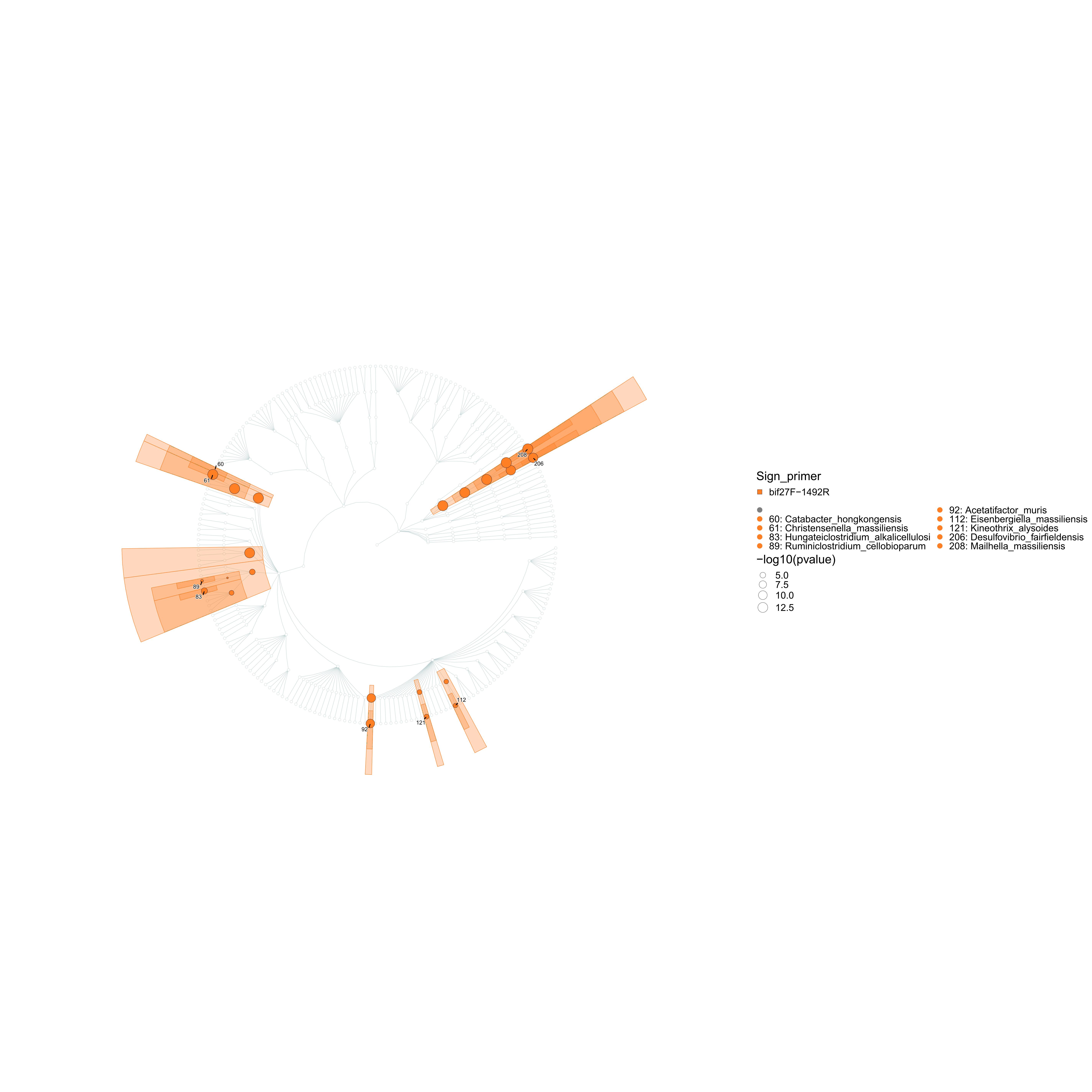

Supplement: SUPPLEMENTARY FIGURE S2 — Bacterial species uniquely identified by the primer combination “bif27F - 1492R” compared to other primers using LefSe. [file Image_2.jpeg]

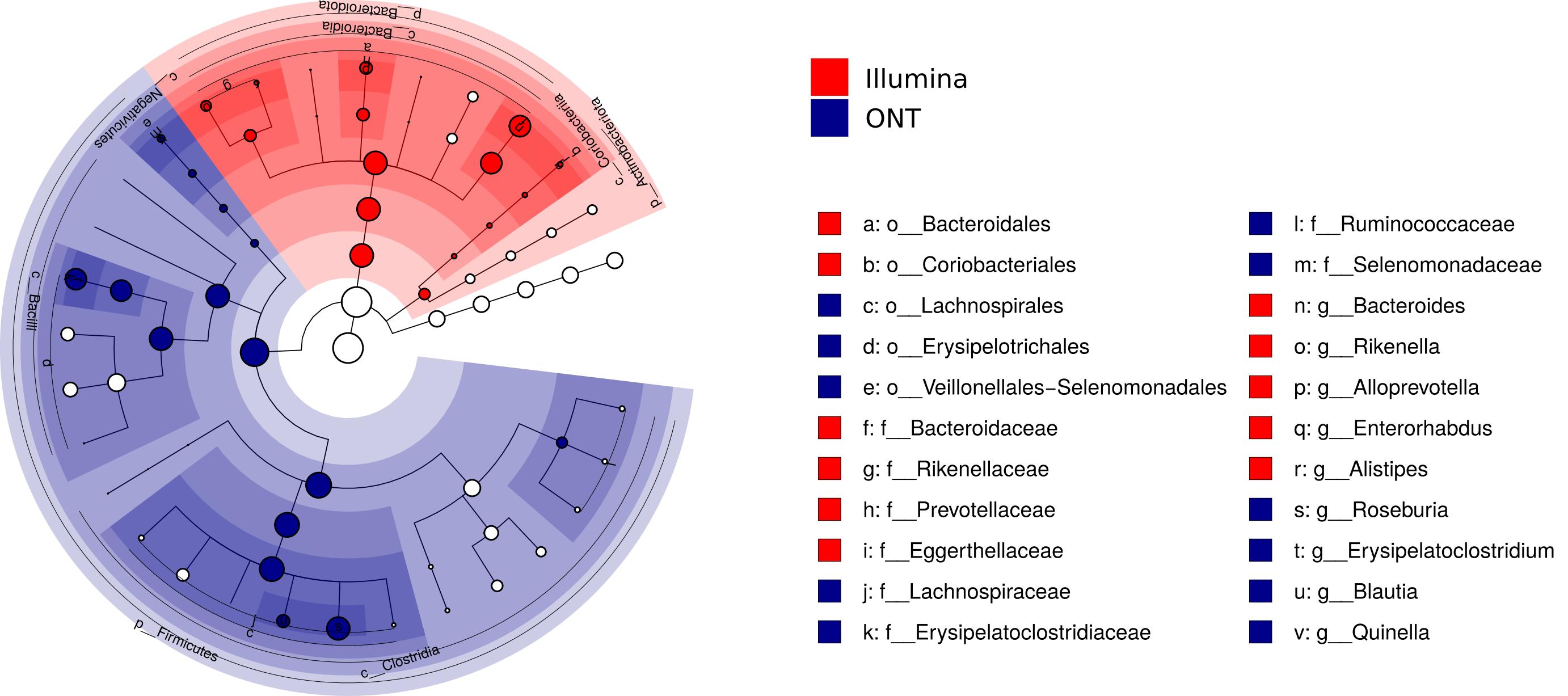

Supplement: SUPPLEMENTARY FIGURE S3 — Differential taxonomic abundance analysis between 16S Illumina and ONT sequencing using LefSe. Red colors represent taxa identified by Illumina 16S rRNA gene sequencing, while blue colors denote taxa identified by ONT sequencing. [file Image_3.jpeg]
